# When ‘Calls for Help’ Backfire: Induced Plant Volatiles Reduce the Attraction of a Nocturnal Predator in Sugarcane

**DOI:** 10.1007/s10886-025-01682-3

**Published:** 2026-01-13

**Authors:** Marvin Pec, Paolo Salazar-Mendoza, Kamila E. X. Azevedo, Diego M. Magalhães, Italo Delalibera, José Maurício S. Bento

**Affiliations:** Department of Entomology and Acarology, Luiz de Queiroz College of Agriculture University of São Paulo, Piracicaba, SP 13418-900 Brazil

**Keywords:** *Doru luteipes*, Earwig, Indirect defense, *Metarhizium robertsii*, Olfactory attraction, *Spodoptera frugiperda*

## Abstract

While herbivore-induced plant volatiles are well-established cues that guide natural enemies to herbivores in ecosystems, microbe-induced plant volatiles have recently gained attention as promising tools for achieving similar outcomes. However, how nocturnal predators respond to volatile cues induced by herbivory and/or endophytic fungal remains poorly understood, particularly in systems where the predator and plant do not share a tightly co-evolved or highly specialized relationship. To explore this, we investigated whether *Spodoptera frugiperda* infestation and *Metarhizium robertsii* endophytic colonization in sugarcane plants could enhance the olfactory attraction of the nocturnal earwig predator *Doru luteipes* by modifying nocturnal volatile emissions and altering endogenous levels of jasmonic acid and salicylic acid. Unexpectedly, the changes in volatile emissions and phytohormone levels induced by herbivory and microbial colonization led to a reduced attraction of the predator compared with undamaged control plants and with the no-plant control. These findings highlight the complexity of *D. luteipes’* responses to induced indirect defenses in sugarcane, suggesting that such strategies may not consistently enhance the recruitment of natural enemies.

**Supplementary Information:**

The online version contains supplementary material available at 10.1007/s10886-025-01682-3.

## Introduction

Since plants form the base of the food chain, they are frequently exposed to herbivore pressure. To cope with this persistent pressure, plants have evolved a range of defensive strategies to reduce damage (Karban and Myers 1989; Mithöfer and Boland 2012). One such strategy is the induction of defenses, which is triggered upon recognition of herbivore attack. This process often involves the transient activation of signaling pathways such as jasmonic acid (JA) and salicylic acid (SA), and the subsequent expression of defensive genes aimed at counteracting the herbivores (Kaplan et al. 2008; Poelman 2015). Induced defenses can extend their effects across multiple trophic levels, producing broader ecological effects. A particularly effective indirect defense involves the attraction of natural enemies, such as predators and parasitoids, that reduce herbivore populations (McCormick et al. 2012; Rodriguez-Saona et al. 2012; Gontijo 2019). This is often mediated by changes in the emission of volatile organic compounds (VOCs), specifically herbivore-induced plant volatiles (HIPVs), which are released in response to herbivory (Dicke and Baldwin 2010). HIPVs act as ‘calls for help’, serving as reliable cues that guide natural enemies to herbivore-infested plants (Dicke 2009; Dicke and Baldwin 2010; Turlings and Erb 2018), thereby contributing to the natural suppression of herbivore populations in crops.

In addition to herbivore-induced defenses, the use of beneficial microbes to enhance plant resistance against herbivores has gained increasing attention in recent years. Certain microbial species can induce systemic resistance in plants (Pieterse et al. 2014; Yan et al. 2019), activating signaling pathways and stimulating the production of secondary metabolites, which can in turn reduce herbivore performance (Schulz et al. 2002; Shikano et al. 2017). Furthermore, microbial inoculation can modify the emission of VOCs, known as microbe-induced plant volatiles (MIPVs) (Sharifi et al. 2018), which influence higher trophic levels by enhancing the attraction of natural enemies (Pappas et al. 2018; Alınç et al. 2024). These findings highlight the potential of integrating microbial approaches into biological control strategies, offering promising improvements for pest management systems.

Sugarcane (*Saccharum officinarum*), a key crop for sugar and bioethanol production worldwide, offers an intriguing model for studying plant indirect defenses mediated by HIPVs and MIPVs. A major pest, the sugarcane borer *Diatraea saccharalis* (Lepidoptera: Crambidae) induces the release of HIPVs when infesting sugarcane stalks, which attract its main natural enemy, the parasitoid *Cotesia flavipes* (Hymenoptera: Braconidae) (Sanches et al. 2017). This parasitoid is widely used in biological control programs targeting *D. saccharalis*. A recent study has shown that the endophytic fungus *Metarhizium robertsii* (Hypocreales: Clavicipitaceae) can colonize aerial sugarcane tissues, resulting in reduced oviposition by *D. saccharalis* (Pec et al. 2024). Moreover, fungal colonization enhances the defensive response of *D. saccharalis*-infested plants, including the JA levels and production of HIPVs, increasing the attraction of *C. flavipes* (Pec et al. 2024). However, sugarcane is extensively cultivated in tropical regions, where it is exposed to a wide array of pests beyond *D. saccharalis*. Among these, the fall armyworm *Spodoptera frugiperda* (Lepidoptera: Noctuidae), a generalist herbivore, feeds on sugarcane leaves, inducing a distinct set of HIPVs (Peñaflor et al. 2017). Interestingly, despite *C. flavipes* not being a typical parasitoid of *S. frugiperda*, the HIPVs emitted by *S. frugiperda*-infested plants can still attract this parasitoid (Peñaflor et al. 2017). Nonetheless, it remains unclear whether nocturnal volatiles induced by herbivore and fungal inoculation can effectively attract predators that are not traditionally employed in sugarcane biological control.

The earwig *Doru luteipes* (Dermaptera: Forficulidae), a Neotropical and predominantly nocturnal predator of several lepidopteran pests, is commonly observed preying on *S. frugiperda* larvae and eggs in South American maize fields (Reis et al. 1988; Silva et al. 2023; Dessie et al. 2024). *Spodoptera frugiperda* is also native to the Americas and is a highly polyphagous herbivore that exploits a wide range of host plants, although maize represent ecologically important and frequently used hosts (Luginbill 1928). The long-standing sympatry between predator and prey in these systems may have favored reliable ecological interactions mediated by plant- and herbivore-derived cues. In maize agroecosystems, *D. luteipes* plays a critical role in pest mortality, significantly contributing to the suppression of *S. frugiperda* populations (Varella et al. 2015). To locate its prey, *D. luteipes* relies on chemical cues, including HIPVs released by maize plants under *S. frugiperda* herbivory at night (Naranjo et al. 2017), suggesting that olfactory-guided foraging plays a crucial role in its predatory behavior. However, while *D. luteipes* is also present in sugarcane fields, its occurrence is less frequent, and its contribution to natural pest control in this crop is limited, with low predation rates documented (Fenoglio and Trumper 2014).

In this study, we investigated the effects of *S. frugiperda* infestation, endophytic colonization by *M. robertsii*, and their combined influence on (*i*) the olfactory attraction of *D. luteipes*, (*ii*) nocturnal plant volatile emissions, and (*iii*) the levels of JA and SA phytohormones in sugarcane. We hypothesized that chemical alterations in sugarcane induced by herbivory and fungal colonization could enhance the plant’s attractiveness to *D. luteipes*. This research aims to elucidate the role of interactions between generalist herbivores and endophytic fungi in shaping sugarcane defense responses and to explore their potential for enhancing biological control strategies.

## Methods and Materials

### Insect Rearing

*Spodoptera frugiperda* and *D. luteipes* were obtained from laboratory colonies originally collected from commercial maize farms in Piracicaba, SP, Brazil. *S. frugiperda* larvae were reared on an artificial diet (Parra 1999) and individualized in glass vials to prevent cannibalism (Chapman et al. 1999). Pupae were placed in plastic containers (20 × 10 × 5 cm), and emerging adults were transferred to cylindrical plastic cages (22 cm high, 10 cm diameter) containing a 10% honey solution. The inner walls of these cages were covered with white paper to facilitate oviposition. Eggs were collected daily, placed in 50-ml plastic cups with artificial diet, and allowed to hatch. Neonates were fed on the diet for 1–2 days before being transferred to glass vials.

Adults and larvae of *D. luteipes* were kept in plastic containers (10 × 24 × 35 cm) covered with organza fabric for ventilation, and lined with brown paper to reduce light exposure. The earwigs were fed ad libitum with an artificial diet (Guimarães 2006), and moistened cotton plugs were provided for hydration. To prevent cannibalism and accommodate their thigmotropic behavior, refuges consisting of accordion-shaped paper and small cardboard boxes (5 × 20 cm) were placed inside the containers. Moistened cotton-filled straw pieces were provided as oviposition substrates (Naranjo-Guevara et al. 2017). The colony has been maintained for approximately 20 generations under laboratory conditions, and to preserve genetic diversity, wild individuals collected from maize fields were introduced every five generations (≈ 200 individuals per introduction). All insect colonies were maintained under controlled conditions (25 ± 2 °C, 60 ± 10% RH, and a L12:D12 photoperiod).

### *Metarhizium robertsii *Fungal Culture

The *M. robertsii* strain ESALQ 1635 was cultured on potato dextrose agar (PDA, Difco Laboratories, Detroit, MI, USA) in Petri dishes, supplemented with Pentabiotic (5 mg/L), and incubated in the dark at 25 °C for 15 days. After eight days, conidia were harvested by suspending them in a 0.05% Tween 80 solution. The surface of the fungal culture was gently scraped using a sterile inoculation spreader (Oliveira et al. 2015). The conidial suspension was then shaken to ensure uniformity and adjusted to a concentration of 1 × 10⁸ conidia mL⁻¹, determined using a Neubauer chamber under a microscope (Axioscop 20; Carl Zeiss, Jena, Germany).

### Plant Growth, Treatments, and Colonization By *Metarhizium robertsii*

The sugarcane variety CTC-9001 was sourced from Grupo São Martinho and cultivated from sets in a greenhouse under natural light and temperature conditions (24 ± 5 °C). Sugarcane seedlings at the tillering phase were grown individually in 1-L plastic pots (17 × 12 × 10 cm) filled with coconut fiber and Basacote^®^ Plus 6 M 16:8:12 (+ 2) fertilizer (Compo, Munster, Germany). At planting, inoculated plants received 1 mL of 1 × 10⁸ conidia mL⁻¹ suspension of *M. robertsii*, applied directly to the soil at the base of each plant, while non-inoculated plants were treated with a 0.05% Tween 80 (v/v) water solution. After nine weeks, a single second-instar *S. frugiperda* larva was placed on each plant to feed for 24 h. The study included four treatments: (*i*) non-inoculated and non-infested plants, (*ii*) *M. robertsii*-inoculated and non-infested plants, (*iii*) non-inoculated and *S. frugiperda*-infested plants, and (*iv*) *M. robertsii*-inoculated and *S. frugiperda*-infested plants. Plants of similar height (~ 45 cm, 9-week-old) were selected for the experiments and subsequent microbiological and chemical analyses.

To confirm endophytic colonization, root and leaf segments (5 cm each) were sterilized through a sequential immersion procedure. Samples were first submerged in 70% ethanol for 1 min, then in 1% sodium hypochlorite for 1 min, and finally in 70% ethanol for 1 min. After sterilization, the fragments were rinsed three times with distilled water and dried on sterile paper. Each fragment was placed individually in Petri dishes (9 × 1.5 cm) containing 15 mL of PDA, supplemented with 0.5 g/L cycloheximide, 0.2 g/L chloramphenicol, 0.5 g/L Dodine (65%), and 0.01 g/L Crystal Violet (Behie et al. 2015). The dishes were incubated at 25 °C in the dark for 12 days. After incubation, the plant segments were examined for fungal mycelial growth under a microscope (Axioscop 20; Carl Zeiss, Jena, Germany).

### Phytohormone Levels

Salicylic acid (SA) and jasmonic acid (JA) levels were measured in leaf tissues from the four treatments (*N* = 12) previously described. Leaf tissues (60–120 mg) were harvested, quickly frozen in liquid nitrogen, and ground to a fine powder. The analysis followed the method described by Schmelz et al. (2003), with adaptations for GC-MS. Each sample was supplemented with 100 ng of dH-JA (Santa Cruz Biotechnology, Santa Cruz, CA, USA) and [2H6] SA (CDN Isotopes, Pointe Claire, QC, Canada) as internal standards for JA and SA, respectively. For extraction, a 400 µL solution was prepared by combining 50 mL of propanol, 25 mL of distilled water, and 50 µL of HCl, followed by the addition of 1 mL of dichloromethane. After centrifugation at 13,000 g for 1 min, 100 µL of a 1:9 diethyl ether solution and 2.3 µL of trimethylsilyl diazomethane (Sigma-Aldrich) were added for derivatization. The reaction was stopped after 25 min by adding 2.3 µL of a 7:3 hexane-acid solutions. Diethyl ether was evaporated, and the samples were heated to 200 °C to remove residual solvent.

Methyl esters of JA and SA were collected by pulling air through an adsorbent filter (30 mg, HayeSep-Q, Alltech Associates, Bannockburn, IL, USA) for 2 min at a flow rate of 1 L min⁻¹. The filters were eluted with 150 µL of dichloromethane, and 2-µL aliquots were analyzed by GC-MS using a QP2010 Ultra system (Shimadzu, Tokyo, Japan) in chemical ionization mode with isobutane as the reagent gas. Phytohormone peaks were identified by monitoring specific ions (SA: 153, [2H6] SA: 157, JA: 225, and dH-JA: 227) and quantified relative to the isotopic standards, corrected for sample weight.

### Headspace Collection and Analysis of Plant Volatiles

Plant volatiles were collected from each treatment (*N* = 9) over a 10-hour period during the nighttime (21:00 to 07:00) under controlled laboratory conditions (25 ± 2 °C, 60 ± 10% RH). Sugarcane plants with their plastic pots and soil wrapped in aluminum foil, were individually placed inside polyethylene terephthalate bags connected to a glass chamber (50 cm width × 36 cm height). The chambers were attached to a volatile collection system (Analytical Research Systems Inc., Gainesville, FL, USA), with filtered and humidified air pumped through at of 0.7 L/min. Volatiles were collected using an adsorbent trap (30 mg, HayeSep Q), connected to the system via PTFE tubing. After collection, the adsorbent traps were eluted with 300 µL of distilled hexane (Sigma Aldrich, St. Louis, MO, USA), and the headspace samples were concentrated to 50 µL under a gentle flow of nitrogen gas.

Volatile analysis was performed on a Shimadzu GC-2010 gas chromatograph, equipped with a non-polar Rtx-1 column (25 mm × 30 m × 25 μm, RESTEK, Bellefonte, PA, USA) and a flame ionization detector (GC-FID) set at 270 °C. The temperature program began at 50 °C for 2 min, followed by a 5 °C/min increase to 180 °C, held for 0.1 min, then ramped at 10 °C/min to 250 °C, and held for 20 min. The injector was maintained at 250 °C. A 10 µL aliquot of a 10 ng/µL nonyl acetate solution (Sigma Aldrich, St. Louis, MO, USA) was added as an internal standard to each sample. A 2-µL aliquot of each sample was injected using a splitless injector with helium as the carrier gas (flow rate: 24 cm/s). Compound quantification was based on peak area comparisons with the internal standard, and results were normalized to dry shoot biomass (g) for each replicate. Data were analyzed using GC Solution software.

For volatile identification, representative samples from each treatment were further analyzed using a Shimadzu GCQP-2010 Ultra quadrupole mass spectrometer, coupled to a Shimadzu GC-2010 gas chromatograph, equipped with a non-polar Rxi-1MS column (25 mm × 30 m × 25 μm, RESTEK, Bellefonte, PA, USA), a splitless injector, and helium as the carrier gas. Ionization was achieved by electron impact (70 eV) with a source temperature of 250 °C. The injector was kept at 250 °C, following the same temperature program as in the GC-FID analysis. Data were processed using GC-MS Solution software. Volatile compounds were identified by comparing their mass spectra with the NIST11 mass spectral library and Kováts retention indices, as well as by injecting authentic standards of the compounds.

### Y-Tube Olfactometer Bioassays

To evaluate the olfactory response of *D. luteipes* to volatiles emitted by treatments, we conducted a series of Y-tube olfactometer assays. The Y-tube olfactometer, with a 3.2 cm diameter and 15 cm length for each arm, was positioned horizontally. Each arm was connected to a cylindrical glass vessel (50 cm in width × 36 cm in height) via Teflon tubing (5 mm in diameter × 40 cm in length). A volatile collection system (Analytical Research System, Gainesville, FL, USA) provided a constant airflow of 1 L/min per arm, directing the plant volatiles from the glass chambers into the olfactometer arms. To avoid visual cues, the glass vessels were positioned behind a black panel, preventing *D. luteipes* from seeing the plants. Experiments were conducted between 20:00 and 23:00 h, during which *D. luteipes* is most active (Naranjo-Guevara et al. 2017), under controlled conditions of 25 ± 2 °C, 70 ± 10% relative humidity, and a 14:10 light-dark photoperiod. Tests were performed in darkness and monitored using an infrared light source.

In each trial, a single female predator (5–15 days old, *N* = 40–45), starved for 48 h prior to the assay, was introduced into the main arm of the olfactometer and allowed up to 5 min to choose between the two arms. A choice was recorded when the female walked at least 8 cm beyond the branching point of the Y-tube, based on direct observation. Females who did not choose within the 5-minute timeframe were excluded from the analysis. Each female was tested only once, and after every ten replicates, the plant combinations were replaced. To avoid positional bias, the odor sources in the chambers were alternated between the left and right arms of the olfactometer, and the Y-tube was cleaned with acetone between trials.

The predator’s choices were evaluated across the following treatment combinations: (*i*) non-inoculated and non-infested plant vs. clean air, (*ii*) *M. robertsii*-inoculated and non-infested plant vs. clean air, (*iii*) *M. robertsii*-inoculated and non-infested plant vs. non-inoculated and non-infested plant, (*iv*) *M. robertsii*-inoculated and *S. frugiperda*-infested plant vs. *M. robertsii*-inoculated and non-infested plant, and (*v*) *M. robertsii*-inoculated and *S. frugiperda*-infested plant vs. non-inoculated and *S. frugiperda*-infested plant.

To confirm that *D. luteipes* responds appropriately to plant volatiles in this sugarcane model, maize was used as a positive control. Previous studies have shown that this predator prefers volatiles from *S. frugiperda*-infested maize plants over those from uninfested plants (Naranjo-Guevara et al. 2017; Pereira et al. 2025). For these assays, a single third-instar *S. frugiperda* caterpillar, starved for 24 h, was used to infest 3-week- old maize plants (hybrid P4285VYHR). The olfactometer tests with maize were conducted under the same experimental conditions (*N* = 30).

### Statistical Analyses

All statistical analyses were performed using R and RStudio Desktop software (https://rstudio.com/). A generalized linear model (GLM) with a Gamma distribution and logit link function was used to assess the effects of treatments on phytohormone levels. For individual volatile compounds, the Kruskal-Wallis test was used, followed by Fisher’s least significant difference for post hoc comparisons. Differences in volatile composition among treatments were examined using permutational multivariate analysis of variance (PERMANOVA). Principal component analysis (PCA) was conducted to visualize treatment-related differences, and a hierarchical clustering heatmap was generated to display variations in the relative abundance of individual volatile compounds. The preference of *D. luteipes* for plant volatiles was analyzed with a GLM using a quasi-binomial distribution and logit link function. The Wald chi-square test was employed to compare responses to different odor sources, and model fit was evaluated with a semi-normal plot within a simulation envelope. The R packages used for these analyses included “lme4” (Bates et al. 2015), “agricolae” (de Mendiburu 2023), “emmeans” (Searle et al. 1980), and “hnp” (Moral et al. 2017).

## Results

### Phytohormone Levels

**Fig. 1:**
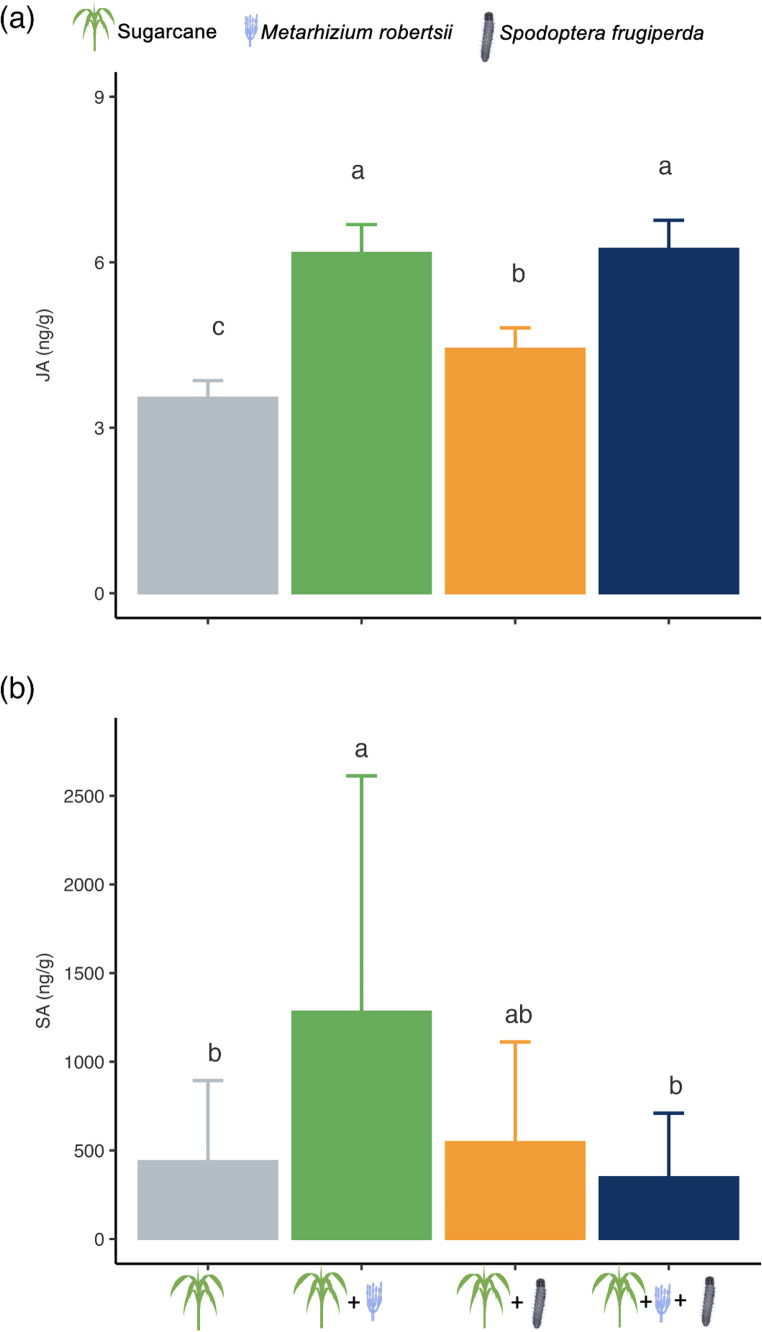
Jasmonic acid – JA (**a**) and salicylic acid – SA (**b**) levels in sugarcane leaves (mean ± SE, ng/g) in non-inoculated plants, plants inoculated with *Metarhizium robertsii*, plants infested with *Spodoptera frugiperda*, and plants both inoculated with *M. robertsii* and infested with *S. frugiperda*. Different letters indicate significant differences among treatments according to Tukey test (*P* < 0.05)

The levels of JA in sugarcane leaves were significantly affected by the treatments (χ² = 26.54, df = 3, *P* < 0.001). Plants inoculated with *M. robertsii*, whether infested by *S. frugiperda* or not, showed significantly higher JA content than plants infested by *S. frugiperda* without fungal inoculation (Fig. 1a). Plants that were neither inoculated nor infested exhibited the lowest JA levels, which were significantly lower than those in *S. frugiperda*-infested plants without inoculation (Fig. 1a). Salicylic acid (SA) content also varied significantly among treatments (χ² = 10.63, df = 3, *P* = 0.0138). Plants inoculated with *M. robertsii* in the absence of herbivory showed significantly higher SA concentrations than both non-inoculated, non-infested plants and plants that were inoculated with *M. robertsii* and infested by *S. frugiperda* (Fig. 1b).

### Plant Volatile Emissions

**Fig. 2:**
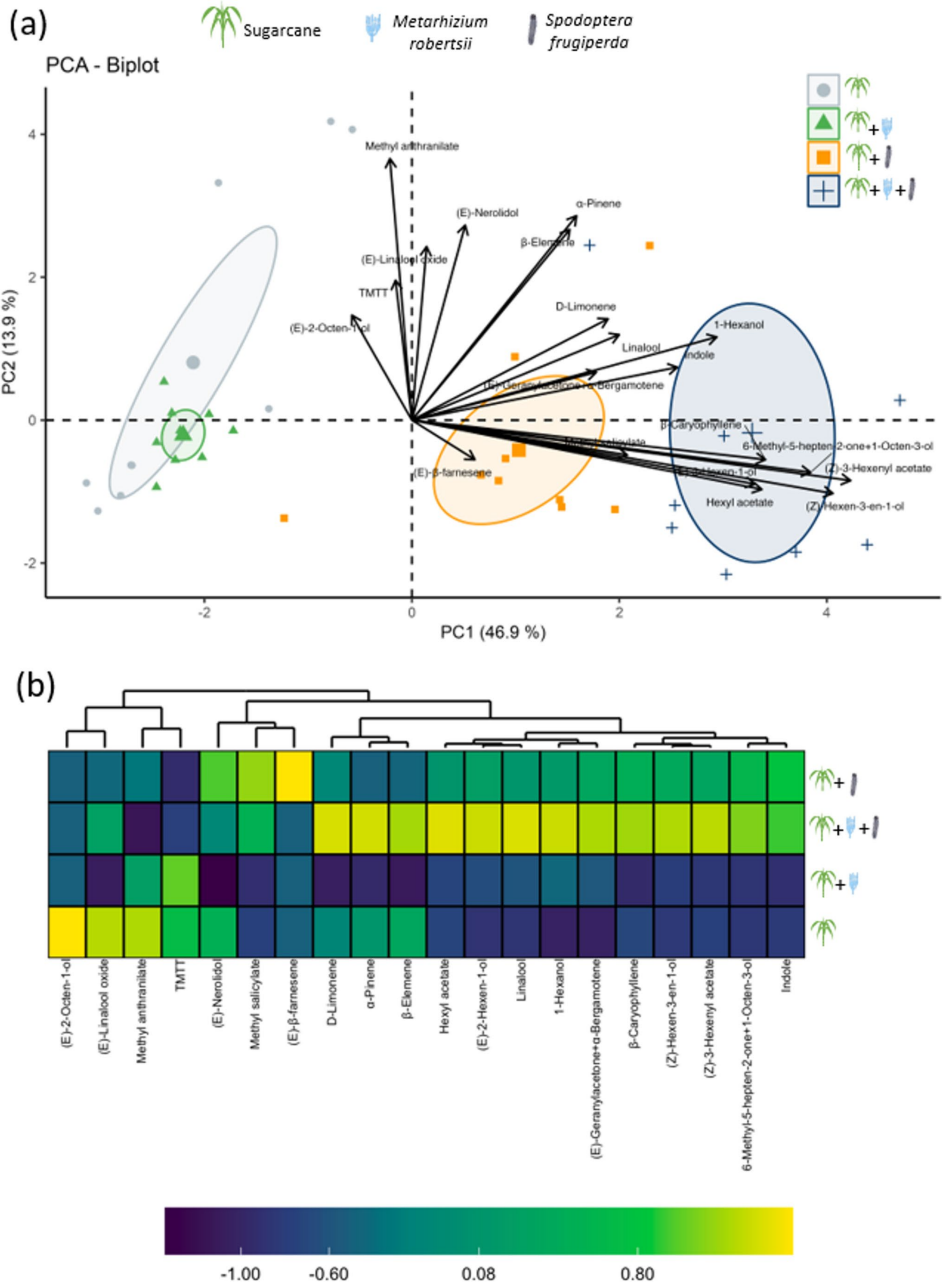
Principal component analysis (**a**) and heatmap plots clusters (**b**) of volatile compounds emitted by non-inoculated sugarcane plants, plants inoculated by *Metarhizium robertsii*, plants with *Spodoptera frugiperda* infestation, and plants both inoculated with *M. robertsii* and infested by *S. frugiperda*

Volatile analysis identified 20 compounds across all treatments, showing both quantitative differences in total emissions and variations in specific compounds. Non-inoculated and non-infested plants emitted 17 detectable compounds, whereas plants inoculated with *M. robertsii* without infestation, plants inoculated with *M. robertsii* and infested with *S. frugiperda*, and non-inoculated plants infested by *S. frugiperda* emitted 14, 18, and 19 compounds, respectively (Table 1). The first two PCA axes accounted for 60.8% of the total variance, revealing differences among treatments based on their volatile profiles (Fig. 2a). Non-inoculated and non-infested plants clustered closely with plants inoculated with *M. robertsii* without herbivory, with the former nearly encompassing the latter. In contrast, plants infested by *S. frugiperda* without inoculation and plants both inoculated and infested were separated from each other and from the other two treatments (Fig. 2a). Plants inoculated with *M. robertsii* and infested by *S. frugiperda* emitted significantly greater total amounts of volatiles than all other treatments, including higher emissions of key compounds such as (*Z*)−3-hexenyl acetate, (*Z*)-hexen-3-en-1-ol, and hexyl acetate (Table 1). Plants infested by *S. frugiperda* without *M. robertsii* inoculation also showed a greater total volatile emission than plants inoculated with *M. robertsii* without infestation and plants that were neither inoculated nor infested. These *S. frugiperda*-infested plants additionally showed higher emission of specific compounds like (*E*)−2-hexen-1-ol, (*Z*)−3-hexenyl acetate, (*Z*)-hexen-3-en-1-ol, 6-methyl-5-hepten-2-one + 1-octen-3-ol, hexyl acetate, and *β*-caryophyllene than *M. robertsii*–inoculated plants without infestation and plants that were neither inoculated nor infested (Table 1). Although plants inoculated with *M. robertsii* without infestation and plants that were neither inoculated nor infested released similar amounts of total volatiles, inoculation with *M. robertsii* appeared to suppress the release of certain compounds, including *β*-caryophyllene, *β*-elemene, and (*E*)-nerolidol (Table 1; Fig. 2b).

**Table 1:** Concentration of volatile organic compounds (mean ± SE, ng/g DW) emitted by sugarcane plants under different treatments: non-inoculated and non-infested (MR-/SF-), inoculated with *Metarhizium robertsii* without infestation (MR+/SF-), non-inoculated and infested with *Spodoptera frugiperda* (MR-/SF+), and both inoculated with *M. robertsii* and infested with *S. frugiperda* (MR+/SF+). Means followed by the same letter do not differ significantly according to fisher’s LSD test (*P* < 0.05)

Number of compounds	Compounds	MR+/SF+				MR-/SF+				MR+/SF-				MR-/SF-				
		x̄	±	SE		x̄	±	SE		x̄	±	SE		x̄	±	SE		*P*
1	(*E*)−2-Hexen-1-ol	3.63	±	0.82	a	1.83	±	0.40	a	0.40	±	0.40	b	0.00	±	0.00	b	**0.0004**
2	(*E*)−2-Octen-1-ol	0.00	±	0.00		0.00	±	0.00		0.00	±	0.00		0.23	±	0.23		0.3916
3	(*E*)-Geranylacetone + α-Bergamotene	2.57	±	0.58	a	1.90	±	0.59	ab	1.15	±	0.44	b	0.77	±	0.24	b	**0.0463**
4	(*E*)-Linalool oxide	0.49	±	0.29		0.31	±	0.31		0.15	±	0.15		0.73	±	0.25		0.1867
5	(*E*)-Nerolidol	0.18	±	0.18		0.35	±	0.23		0.00	±	0.00		0.28	±	0.19		0.4968
6	(*E*)-*β*-Farnesene	0.00	±	0.00		0.07	±	0.07		0.00	±	0.00		0.00	±	0.00		0.3916
7	(*Z*)−3-Hexenyl acetate	57.10	±	3.72	a	32.75	±	4.15	b	1.73	±	0.65	c	3.66	±	1.03	c	**0.0001**
8	(*Z*)-Hexen-3-en-1-ol	60.82	±	6.88	a	36.90	±	3.42	b	1.79	±	0.47	c	0.70	±	0.20	d	**0.0001**
9	1-Hexanol	3.71	±	0.40	a	2.54	±	0.48	ab	1.93	±	0.62	b	1.33	±	0.51	b	**0.0194**
10	6-Methyl-5-hepten-2-one + 1-Octen-3-ol	21.07	±	2.11	a	16.48	±	1.82	a	1.62	±	0.36	b	1.86	±	0.27	b	**0.0001**
11	D-Limonene	1.81	±	0.68	a	0.98	±	0.47	ab	0.47	±	0.29	b	0.97	±	0.41	ab	**0.0457**
12	Hexyl acetate	1.25	±	0.26	a	0.48	±	0.22	b	0.00	±	0.00	c	0.00	±	0.00	c	**0.0003**
13	Indole	6.02	±	1.31	a	5.76	±	2.01	ab	1.62	±	0.47	ab	1.82	±	0.98	b	**0.0360**
14	Linalool	23.18	±	2.18	a	18.59	±	1.29	ab	16.13	±	3.11	b	15.14	±	1.43	b	**0.0430**
15	Methyl anthranilate	0.46	±	0.46		0.72	±	0.40		0.90	±	0.28		1.20	±	0.45		0.2549
16	Methyl salicylate	2.23	±	0.55	a	2.90	±	0.76	a	0.63	±	0.27	b	0.80	±	0.41	b	**0.0215**
17	*β*-Caryophyllene	9.01	±	2.06	a	6.08	±	1.23	a	0.00	±	0.00	b	1.03	±	0.64	b	**0.0005**
18	*β*-Elemene	1.01	±	0.53		0.28	±	0.28		0.00	±	0.00		0.65	±	0.33		0.2103
19	TMTT	0.16	±	0.07		0.15	±	0.09		0.27	±	0.24		0.25	±	0.17		0.8386
20	α-Pinene	1.33	±	0.46		0.57	±	0.24		0.38	±	0.25		0.83	±	0.42		0.3951
	**Total**	196.03	**±**	8.96	**a**	129.63	**±**	8.82	**b**	29.17	**±**	2.63	**c**	32.24	**±**	5.56	**c**	**0.0001**

Numbers in bold indicate significant effects at α = 0.05

### Olfactory Attraction of *Doru luteipes*

**Fig. 3:**
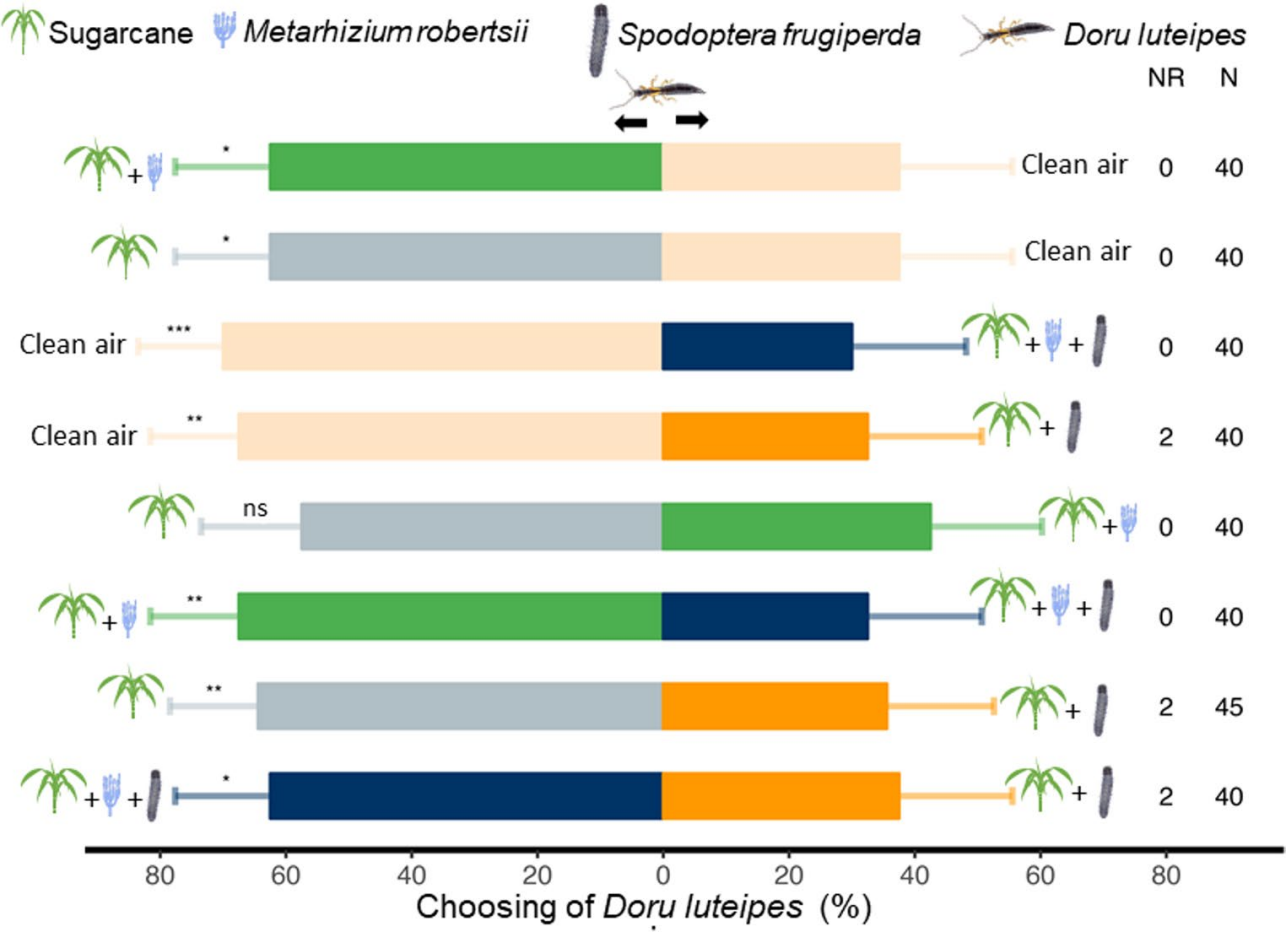
Responses of *Doru luteipes* females to the volatiles emitted by sugarcane plants in a Y-tube olfactometer. Choices tested consisted of: *Metarhizium robertsii*-inoculated plants vs. clean air, non-inoculated and non-infested plants vs. clean air, clean air vs. *M. robertsii*-inoculated plants infested by *Spodoptera frugiperda*, clean air vs. plants infested by *S. frugiperda*, non-inoculated and non-infested plants vs. *M. robertsii*-inoculated plants, *M. robertsii*-inoculated plants vs. *M. robertsii*-inoculated plants infested by *S. frugiperda*, non-inoculated and non-infested plants vs. non-inoculated plants infested by *S. frugiperda*, and *M. robertsii*-inoculated plants infested by *S. frugiperda* vs. non-inoculated plants infested by *S. frugiperda.* *P ≤ 0.05; **P ≤ 0.01; ***P ≤ 0.001; ns *P* > 0.05. N = number of responses of *D. luteipes* in each choice test, NR = number of non-responses in each choice test

*Doru luteipes* was more attracted to volatiles emitted by plants inoculated with *M. robertsii* and uninfested than to clean air (χ² = 4.89, df = 1, *P* = 0.0269). The predator also preferred volatiles from uninoculated and uninfested plants over clean air (χ² = 4.89, df = 1, *P* = 0.0269). Conversely, *D. luteipes* showed a strong preference for clean air over volatiles emitted by plants inoculated with *M. robertsii* and infested by *S. frugiperda* (χ² = 12.061, df = 1, *P* < 0.001), as well as over plants infested by *S. frugiperda* without fungal inoculation (χ² = 9.37, df = 1, *P* = 0.0021) (Fig. 3). Consistent with these results, *D. luteipes* was more attracted to plants inoculated with *M. robertsii* and uninfested than to plants inoculated and infested (χ² = 9.37, df = 1, *P* = 0.0021), and more attracted to uninoculated and uninfested plants than to uninoculated but infested plants (χ² = 7.29, df = 1, *P* = 0.0069). No significant difference was detected between the predator’s attraction to plants inoculated with *M. robertsii* and uninfested and uninoculated and uninfested plants (χ² = 1.78, df = 1, *P* = 0.1814). Finally, plants inoculated with *M. robertsii* and infested were more attractive than those infested without inoculation (χ² = 4.89, df = 1, *P* = 0.0269) (Fig. 3).

In the positive control assays, *D. luteipes* females were significantly more attracted to volatiles emitted by *S. frugiperda*-infested maize plants than to those from uninfested plants (χ² = 4.17, df = 1, *P* = 0.047) (Supplemental Fig. 1).

## Discussion

In a previous study, we demonstrated the key role of *M. robertsii* in increasing JA levels in sugarcane upon inoculation, with these levels further enhanced in the presence of *D. saccharalis* infestation (Pec et al. 2024). In this current study, we show that *M. robertsii-*inoculated sugarcane consistently increased JA content, regardless of *S. frugiperda* infestation (Fig. 1A). This suggests that *M. robertsii* may strengthen the plant’s defense system by activating the JA pathway, potentially priming the plant for a more robust response to herbivores compared to the JA response triggered solely by *S. frugiperda*. Recent studies in maize support this finding, showing that *M. robertsii* inoculation leads in higher expression of key enzymes involved in JA biosynthesis, such as lipoxygenase and 12-oxo-phytodienoate reductase 7 (Ahmad et al. 2020; Peterson et al. 2023). Additionally, certain strains of *Metarhizium anisopliae* (Hypocreales: Clavicipitaceae) have been shown to increase JA content in both leaves and roots of maize (Rivas-Franco et al. 2020).

Salicylic acid pathways are also noteworthy in the context of plant defenses against biotic stressors such as pathogens. While previous studies in maize have shown that *M. robertsii* inoculation does not alter SA content (Ahmad et al. 2020), our study indicates that sugarcane plants inoculated with *M. robertsii* exhibited higher SA levels than non-inoculated plants (Fig. 1B). However, SA concentrations decreased in inoculated plants when they were infested by *S. frugiperda*. This pattern mirrors what we previously observed in plants infested by *D. saccharalis* (Pec et al. 2024). Since *M. robertsii* activated both SA and JA pathways, future research should investigate its potential to provide dual defense against both chewing herbivores and pathogenic fungi in sugarcane.

In response to changes in their defense pathways, plants may adjust the types or amounts of volatile compounds they emit (Ponzio et al. 2013; Wei et al. 2014). Induced defenses via HIPVs and, more recently, MIPVs are considered promising cues for attracting natural enemies and reducing pest damage (Sharifi et al. 2018; Thomas et al. 2023). Although it may seem obvious that natural enemies would respond to these volatiles, it is still uncertain whether they react similarly under different conditions or in response to specific contexts. In this study, we demonstrate that the generalist predator *D. luteipes* is attracted to sugarcane plants irrespective of *M. robertsii* inoculation, but exhibits reduced attraction toward plants infested by *S. frugiperda*, regardless of the presence of *M. robertsii*, when compared with clear air.

While total emission levels remained similar between inoculated and non-inoculated sugarcane plants, *M. robertsii* suppressed specific compounds, including (*E*)-nerolidol, *β*-caryophyllene, and *β*-elemene. Despite these differences, *D. luteipes* showed no preference between inoculated and non-inoculated plants when choosing between their volatiles, though it was attracted to both over clean air. This suggests that the observed volatile variations may not significantly influence the predator’s attraction in sugarcane. While there is growing interest in the potential of MIPVs to attract natural enemies, responses to these blends have been inconsistent across studies. For instance, the predator *Macrolophus basicornis* (Hemiptera: Miridae) was more attracted to tomato plants inoculated with *M. robertsii* (Salazar-Mendoza et al. 2025), whereas inoculations with *Metarhizium brunneum* (Hypocreales: Clavicipitaceae), *Beauveria bassiana* (Hypocreales: Cordycipitaceae), and *Trichoderma harzianum* (Hypocreales: Hypocreaceae) did not enhance the attraction of *Macrolophus pygmaeus* and *Nesidiocoris tenuis* (Hemiptera: Miridae) (Meesters et al. 2024). Similarly, *M. robertsii* inoculation in sugarcane did not enhance the response of *C. flavipes*, despite the fungus’s effect on volatile emissions (Pec et al. 2024). These discrepancies underscore the need for further research to better understand the complex role of MIPVs in predator-prey interactions and their potential to enhance biological control strategies. On the other hand, our previous study showed that *M. robertsii* suppressed diurnal emissions of (*E*)-nerolidol and three other compounds (Pec et al. 2024). Interestingly, in the current study, we found that *M. robertsii* inoculation reduced nocturnal emissions of different compounds compared to diurnal emissions, suggesting that the fungus may influence circadian-regulated volatile emissions. This could potentially alter plant interactions with herbivores and natural enemies that are active at night.

The HIPVs are reliable cues that natural enemies use to locate insect herbivores (Turlings et al. 1998; Dicke and Baldwin 2010; McCormick et al. 2012). A previous study has shown *S. frugiperda* infestation alters volatile profiles, leading to an overall increase in emissions, particularly of compounds like (*E*)−2-hexen-1-ol, 6-methyl-5-hepten-2-one + 1-octen-3-ol, and *β*-caryophyllene (Peñaflor et al. 2017), findings which align with our results. Additionally, our study reveals that *M. robertsii* inoculation further enhances the total emission of HIPVs in *S. frugiperda*-infested sugarcane, particularly for specific compounds such as (*Z*)−3-hexenyl acetate, hexyl acetate, and (*Z*)-hexen-3-en-1-ol. These changes could potentially bolster indirect plant defenses by attracting natural enemies. As observed previously, combined *D. saccharalis* infestation and *M. robertsii* inoculation increased the attraction of *C. flavipes* beyond the effect of HIPVs alone (Pec et al. 2024). However, contrary to our expectations, *D. luteipes* avoided plants with high volatile emissions in both treatments and even compared to clean air. This suggests that volatile cues produced by sugarcane in these specific treatments are either repellent or, more likely, lack the specific attractant signals *D. luteipes* uses for hunting. This stands in contrast to its known attraction to nocturnally emitted HIPVs from maize plants infested by *D. saccharalis* or *S. frugiperda* (Naranjo et al. 2017). We hypothesize that *D. luteipes* exhibits a stronger behavioral responsiveness to volatile blends characteristic of maize, likely reflecting ecological familiarity or sensory bias toward a crop frequently associated with suitable prey. In contrast, the distinct chemical composition of sugarcane volatiles, influenced by *S. frugiperda* infestation alone or combined with *M. robertsii* inoculation, may disrupt this association, making sugarcane less effective at attracting *D. luteipes*. Indeed, plant species specificity is crucial in shaping natural enemy-plant interactions through HIPVs (Takabayashi 2022), and plant genotypes can also influence these variations (Russavage et al. 2024).

In summary, although *M. robertsii* inoculation increased JA and SA levels in sugarcane and altered its nocturnal volatile profile, it did not enhance the attraction of the predator *D. luteipes*. Likewise, *S. frugiperda* infestation, with or without *M. robertsii*, increased JA levels and volatile emissions but reduced predator attraction. These findings reveal the complex and sometimes unpredictable responses of natural enemies to plant volatiles, suggesting that the ecological outcomes of plant defenses are context-dependent and may not always be effective in recruiting natural enemies. It is important to note that our conclusions are constrained by the specific experimental conditions employed, including plant genotype, plant age, single fungal inoculation, and infestation intensity. Future studies should incorporate field-based approaches to better capture the complexity of volatile-mediated interactions under more realistic conditions in sugarcane agroecosystems.

## Supplementary Information

Below is the link to the electronic supplementary material.

ESM 1 (DOCX 50.9 KB)

## Data Availability

No datasets were generated or analysed during the current study. The data supporting this study are available upon reasonable request.
