# Supplementary material for: When ‘Calls for Help’ Backfire: Induced Plant Volatiles Reduce the Attraction of a Nocturnal Predator in Sugarcane
Source: J Chem Ecol. 2026 Jan 13;52(1):7. doi: 10.1007/s10886-025-01682-3 (PMC12799659; doi:10.1007/s10886-025-01682-3)

**When ‘Calls for Help’ Backfire: Induced Plant Volatiles Deter the Attraction of a Nocturnal Predator in Sugarcane**

**Supplemental Figure 1.** Responses of *Doru luteipes* females to volatiles emitted by maize plants infested by *Spodoptera frugiperda* compared to uninfested maize plants, assessed in a Y-tube olfactometer. **P* ≤ 0.05; NR = number of non-responses in each choice test; N = number of responses of *D. luteipes* in each choice test.


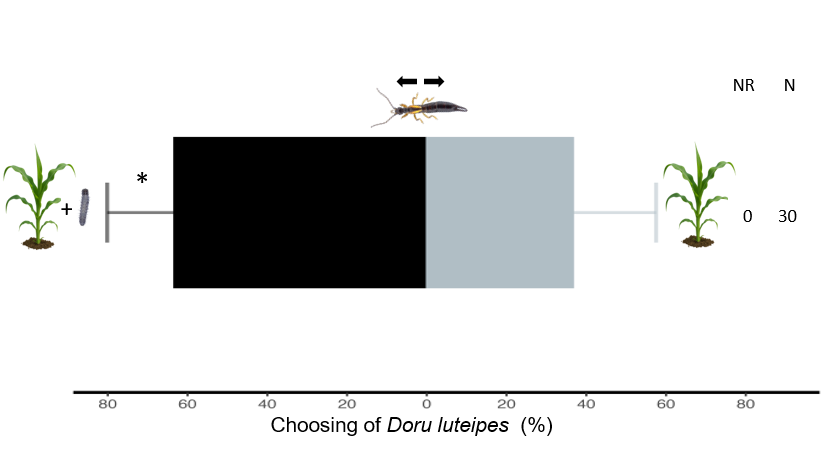

Supplement: Supplementary file 1 — (DOCX 50.9 KB) [file 10886_2025_1682_MOESM1_ESM.docx]
